# Updating Molecular Diagnostics for Detecting Methicillin-Susceptible and Methicillin-Resistant Staphylococcus aureus Isolates in Blood Culture Bottles

**DOI:** 10.1128/JCM.01195-19

**Published:** 2019-10-23

**Authors:** Fred C. Tenover, Isabella A. Tickler, Victoria M. Le, Scott Dewell, Rodrigo E. Mendes, Richard V. Goering

**Affiliations:** a Cepheid, Sunnyvale, California, USA; b JMI Laboratories, North Liberty, Iowa, USA; c Creighton University, Omaha, Nebraska, USA; bioMérieux

**Keywords:** MRSA, SCCmec, oxacillin resistance, empty cassette

## Abstract

Molecular diagnostic tests can be used to provide rapid identification of staphylococcal species in blood culture bottles to help improve antimicrobial stewardship. However, alterations in the target nucleic acid sequences of the microorganisms or their antimicrobial resistance genes can lead to false-negative results. We determined the whole-genome sequences of 4 blood culture isolates of Staphylococcus aureus and 2 control organisms to understand the genetic basis of genotype-phenotype discrepancies when using the Xpert MRSA/SA BC test (*in vitro* diagnostic medical device [IVD]). Three methicillin-resistant S. aureus (MRSA) isolates each had a different insertion of a genetic element in the staphylococcal cassette chromosome (SCC*mec*)-*orfX* junction region that led to a misclassification as methicillin-susceptible S. aureus (MSSA). One strain contained a deletion in *spa*, which produced a false S. aureus-negative result. A control strain of S. aureus that harbored an SCC*mec* element but no *mecA* (an empty cassette) was correctly called MSSA by the Xpert test. The second control contained an SCC_M1_ insertion. The updated Xpert MRSA/SA BC test successfully detected both *spa* and SCC*mec* variants of MRSA and correctly identified empty-cassette strains of S. aureus as MSSA. Among a sample of 252 MSSA isolates from the United States and Europe, 3.9% contained empty SCC*mec* cassettes, 1.6% carried SCC_M1_, <1% had *spa* deletions, and <1% contained SCC*mec* variants other than those with SCC_M1_. These data suggest that genetic variations that may interfere with Xpert MRSA/SA BC test results remain rare. Results for all the isolates were correct when tested with the updated assay.

## Abstract (precis)

Molecular diagnostic tests can be used to provide rapid identification of staphylococcal species in blood culture bottles to help improve antimicrobial stewardship. However, alterations in the target nucleic acid sequences of the microorganisms or their antimicrobial resistance genes can lead to false-negative results.

## INTRODUCTION

Staphylococcus aureus and methicillin-resistant S. aureus (MRSA) continue to be leading causes of bloodstream infections (BSI) (1). Molecular diagnostic tests to identify the presence of methicillin-susceptible S. aureus (MSSA) and MRSA isolates in clinical specimens, including blood culture bottles and wounds, are being used with increasing frequency to guide antimicrobial therapy for staphylococcal infections (2, 3). The results of molecular diagnostic tests, such as those that employ PCR or other nucleic acid amplification strategies, can aid antimicrobial stewardship efforts (4, 5). However, results reported by molecular tests can be confounded by changes in target nucleic acid sequences. This is especially true with pathogens, such as S. aureus, for which 15 to 20% of the genome may contain mobile genetic elements (MGE) (6). MGE often carry antimicrobial resistance genes or virulence determinants and can insert into, or adjacent to, staphylococcal cassette chromosome *mec* (SCC*mec*) elements, altering the target of a molecular test and sometimes the organism’s phenotype (7, 8). Differences between the results of phenotypic and genotypic tests reported by the laboratory for blood cultures can be confusing for physicians and can affect therapeutic regimens.

In this study, we determined the whole-genome sequences of four blood culture isolates and two control strains of S. aureus to understand the genetic basis of the discrepancies observed between the genotype of the isolates determined by the Xpert MRSA/SA BC test (Cepheid, Sunnyvale, CA), which received FDA clearance in June 2013 (here referred to as Xpert MRSA/SA BC 2013), and the phenotypic results of antimicrobial susceptibility tests. We then obtained a convenience sample of MSSA isolates collected from laboratories in the United States and Europe to determine the prevalence of the mobile elements, such as SCC_M1_, and genetic changes, such as empty cassettes and *spa* mutations, that may affect PCR results. These results were compared with those of an updated version of the Xpert MRSA/SA BC test, which received FDA clearance in June 2019 (here referred to as Xpert MRSA/SA BC 2019).

## MATERIALS AND METHODS

### Bacterial strains.

The S. aureus isolates used in the study are listed in Table 1, along with their U.S. state of origin, oxacillin and cefoxitin susceptibility test results, and other resistance genes identified from whole-genome sequencing (WGS). Isolates were selected from a collection of 30 S. aureus strains investigated over the last 4 years that demonstrated phenotype-genotype discrepancies between Xpert MRSA/SA BC test results and the results of phenotypic susceptibility testing. The most common insertion elements noted from DNA sequence analysis were included in this study (e.g., SCC_M1_ represented approximately one-third of the elements identified). Organisms were identified using Gram stain, catalase, and coagulase testing and Pos ID type 3 MicroScan WalkAway identification panels (Beckman Coulter, Brea, CA). Antimicrobial susceptibility testing was performed using the MicroScan Walkaway Pos MIC panel type 29 (Beckman Coulter) according to the manufacturer’s instructions. The isolates were also tested using the disk diffusion method according to Clinical and Laboratory Standards Institute (CLSI) guidelines (9) using both cefoxitin and oxacillin disks and interpreted using CLSI document M100, the 28th edition for cefoxitin (10) and the 22nd edition for oxacillin (11). A cefoxitin induction test was performed by inoculating a Mueller-Hinton plate with a 0.5 McFarland suspension of the organism, placing a 30-μg cefoxitin disk in the middle of the plate, and incubating the plate overnight at 35°C. Growth was taken from the inner edge of the zone of inhibition and used to prepare the inoculum for a second disk diffusion test. Colonies within the zone of inhibition were tested by MicroScan MIC panels to confirm oxacillin resistance. Quality control organisms for antimicrobial susceptibility testing included S. aureus ATCC 29213, S. aureus ATCC 25923, S. aureus ATCC 43300, S. aureus ATCC BAA-977, Enterococcus faecalis ATCC 29212, and Escherichia coli ATCC 35218.

**TABLE 1:** AST results and resistance genes identified in S. aureus isolates^*a*^

Isolate (state)	AST result^*b*^				Antimicrobial resistance gene(s)				
	Cefoxitin ZOI (mm)	Oxacillin ZOI (mm)	Cefoxitin screen (μg/ml)	Oxacillin MIC (μg/ml)	Aminoglycoside	Beta-lactam	Fluoroquinolone	Trimethoprim	Macrolide, lincosamide, streptogramin B
16439 (MA)	6	6	>4	>2	*spc*, *aadD*	*blaZ*, *mecA*	*norA*		*msr*(A), *erm*(A), *mph*(C)
16445 (ME)	24 (8)	6 (with haze)	≤4 (>4)	>2	*aadD*	*blaZ*, *mecA*	*norA*	*dfrG*	*msr*(A), *mph*(C)
15100 (NC)	24 (18)	18 (6 with haze)	>4	0.5 (>2)	*spc*, *aadD*	*mecA*	*norA*		*erm*(A)*, erm*(C)
16514 (KS)	16	6	>4	>2	*aph(3')-III*, *ant(6)-Ia*	*blaZ*, *mecA*	*norA*		*msr*(A), *mph*(C)
15077 (OR)	25	13	≤4	0.5	*aph(3')-III*, *ant(6)-Ia*, *spc*	*blaZ*	*norA*		*msr*(A), *mph*(C)
15050 (WA)	25	17	≤4	≤0.25	*spc*	*blaZ*	*norA*		*erm*(A)

a AST, antimicrobial susceptibility testing; ZOI, zone of inhibition measured with disk diffusion testing.

b Results in parentheses were obtained after exposure to cefoxitin (i.e., induction testing).

### PCR.

A 50-μl aliquot from a positive blood culture bottle showing Gram-positive cocci in clusters was tested using the Xpert MRSA/SA BC 2013 test (*in vitro* diagnostic medical device [IVD]) (Xpert MRSA/SA BC 2013; Cepheid, Sunnyvale, CA) as described by the manufacturer. The test has three targets: the gene encoding staphylococcal protein A (*spa*), the methicillin resistance gene *mecA* (*mec*), and the junction region between *orfX* in the S. aureus chromosome and the SCC*mec* element. In this version of the assay, all three targets must be positive for a result of MRSA to be reported. However, S. aureus is reported as positive if *spa* is positive whether or not any other targets are positive. In the updated version of the Xpert MRSA/SA BC 2013 test, rule-based algorithms are applied to the results of the three targets to differentiate between MSSA and MRSA. Under the rule-based algorithms, MRSA isolates that are positive only for the *spa* and *mec* targets, or positive for *mec* and SCC*mec* targets, are reported as MRSA if the conditions of the rules are met. There are no changes to the probes, primers, buffers, amplification conditions, or intended use in the Xpert MRSA/SA BC 2019 test. The isolates were tested with both the former and updated versions of the Xpert MRSA/SA BC test. Cycle threshold (*C_T_*) values for the *spa*, *mec*, and SCC*mec* targets were used to identify potential *spa* variants, empty-cassette strains (*spa*^+^, *mec* negative, and SCC*mec*^+^), and SCC*mec* variants (*spa*^+^, *mec*^+^, and SCC*mec* negative), which were confirmed by DNA sequence analysis (see below). Quality control organisms for PCR included S. aureus ATCC 25923 (MSSA) and S. aureus ATCC 43300 (MRSA; SCC*mec* type II).

### Whole-genome sequencing and analysis.

Genetic sequencing was undertaken with pure cultures of S. aureus grown overnight at 35°C in tryptic soy broth (Hardy Diagnostics, Santa Maria, CA). Nucleic acid was extracted from the broth cultures using the Sigma-Aldrich (St. Louis, MO) GenElute bacterial genomic DNA kit according to the manufacturer’s instructions. Concentrations of DNA were determined by the UV light absorbance method using the NanoPhotometer system (Implen, Munich, Germany). Sequencing libraries were prepared from extracted genomic DNA using a Nextera XT (Illumina, San Diego, CA) kit and rapid barcoding kit (Oxford Nanopore Technologies, Oxford, United Kingdom). Libraries were quantified with a Qubit 4 fluorometer, using a double-stranded DNA (dsDNA) high-sensitivity assay kit (Invitrogen, Carlsbad, CA). Resultant libraries were sequenced on both short-read and long-read sequencing platforms, accordingly. Libraries prepared with the Nextera kit were sequenced on the MiSeq (Illumina) using V3 reagent chemistry with 301-cycle paired-end reads. Libraries prepared with the rapid barcoding kit were sequenced on the MinION (Oxford Nanopore Technologies) using flow cell R9.4.1. Hybrid assemblies were generated from short- and long-read fastq files using Unicycler v0.4.6 (12), a software pipeline that performs a series of operations that include adapter trimming, quality control, error correction, assembly, and scaffolding. The software was used with default settings. Genomes were annotated using prokka 1.12 (13) and analyzed using Center for Genomic Epidemiology (CGE) online tools (14), SnapGene Viewer (GSL Biotech, snapgene.com), and BioNumerics v7.6 (Applied Maths, Sint-Martens-Latem, Belgium).

### Surveillance study.

One hundred fifty-two phenotypically MSSA isolates collected from hospitalized patients in the United States in 2016, and 100 isolates of MSSA from hospitalized patients in Europe in 2017, were obtained from JMI Laboratories (North Liberty, IA), focusing on prevalence of SCC_M1_, *spa* variants, and empty cassettes, as indicated by analysis of the 30 discrepant isolates. These isolates were part of the SENTRY Antimicrobial Surveillance Program. Organisms were identified as S. aureus as previously described (15). Isolates were tested initially with the Xpert MRSA/SA BC 2013 test using 50 μl of a 0.5 McFarland suspension of colonies in pure culture prepared in MicroScan sterile inoculum water (Beckman Coulter). (This is considered off-label testing.) Isolates were tested for the presence of the *spa*, *mec*, and SCC*mec* targets and then screened with the following two sets of PCR primers specific for SCC_M1_/SCC_266_ elements: 5′-TACGATTTTGAGCTAGCTTTTCG-3′ and 5′-ATTTTCGTTCGATCGGGGGT-3′ (2.4-kb product at 58°C) and 5′-CTCCAGAACTAAGATTTCCAGAGT-3′ and 5′-GGGTTTCACTCGAATGTCCGTA-3′ (1.4-kb product at 58°C). Isolates were also tested using the Xpert MRSA/SA BC 2019 test.

### Accession number(s).

Accession numbers for the sequences described can be found under NCBI BioProject accession number PRJNA555368.

## RESULTS

**FIG 1:**
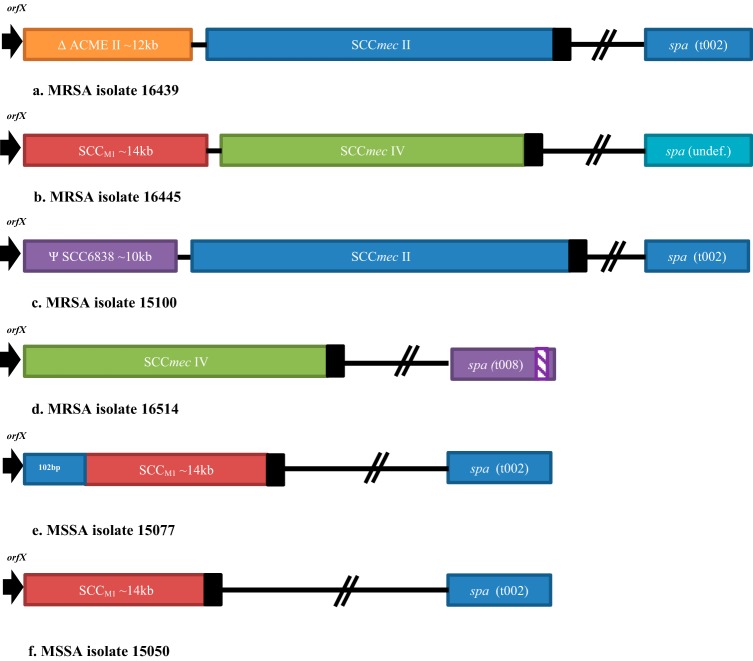
Schematic diagram showing the genomic alterations identified in the six S. aureus isolates characterized in this study (not to scale): MRSA isolate 16439 with ACME II inserted between *orfX* and SCC*mec* II at the integration site *attB* at the 3′ end of *orfX* (*attB*_SCC_) (a), MRSA isolate 16445 with SCC_M1_ element inserted between *orfX* and SCC*mec* IV at *attB*_SCC_ (b), MRSA isolate 15100 with a pseudo-SCC_6838_ element inserted between *orfX* and SCC*mec* II at *attB*_SCC_ (c), MRSA isolate 16514 with a deletion of 23 bp in the *spa* gene (d), MSSA isolate 15077 with remnants of SCC*mec* and an SCC_M1_ element inserted at a second *attB* site, 84 bp downstream of the 3′ end of *orfX*, but missing *mecA* (empty cassette strain) (e), and MSSA isolate 15050 with SCC_M1_ element, inserted at *attB*_SCC_, but no SCC*mec* remnants, for comparison with isolate 15077 (f).

The isolate characteristics, PCR cycle threshold (*C_T_*) values obtained when tested with the Xpert MRSA/SA BC 2013 test, and interpretations with the updated Xpert MRSA/SA BC 2019 test, rule-based algorithms are shown in Table 2. A schematic of the genetic alterations observed by WGS in the six S. aureus isolates is shown in Fig. 1.

**TABLE 2:** PCR results, genotypes, and genetic alterations identified in S. aureus isolates

Isolate	Typing/WGS result				PCR cycle threshold value			Xpert MRSA/SA BC 2013 result	Xpert MRSA/SA BC 2019 result
	MLST^*a*^	*spa*/SCC*mec* type	Additional element(s) identified^*b*^	Genetic alteration	*spa*	*mec*	SCC*mec*		
16439	3390	t002/II(2A)	None	ACME insertion in *orfX*	16.3	16.4	0	MRSA negative, S. aureus positive	MRSA positive, S. aureus positive
16445	8	Undefined/IV(2B)	*ccr* class 4	SCC_M1_ insertion in *orfX*	17.5	17.6	0	MRSA negative, S. aureus positive	MRSA positive, S. aureus positive
15100	5	t002/II(2A)	*ccr* class 5	ΨSCC_6838_ insertion in *orfX*	17.7	17.8	0	MRSA negative, S. aureus positive	MRSA positive, S. aureus positive
16514	8	t008/IVa(2B)	None	23-bp deletion in *spa*	0	14.7	15.9	MRSA negative, S. aureus negative	MRSA positive, S. aureus positive
15077	5	t002/none	*ccr* class 4	Deletion of *mecA* empty cassette, SCC_M1_ insertion in *orfX*	21.1	0	22.6	MRSA negative, S. aureus positive	MRSA negative, S. aureus positive
15050	5	t002/none	*ccr* class 4	SCC_M1_ insertion in *orfX*	18.2	0	0	MRSA negative, S. aureus positive	MRSA negative, S. aureus positive

a MLST, multilocus sequence type.

b Results obtained with CGE SCC*mec*Finder.

### SCC insertion element 1: ACME.

Results for the Xpert MRSA/SA BC 2013 test performed on a positive blood culture bottle initially were reported as MRSA negative and S. aureus positive. However, the isolate (16439) recovered from the bottle was phenotypically MRSA by both MIC and disk diffusion testing (Table 1). The *C_T_* values for the test were 16.3 for *spa* (positive), 16.4 for *mec* (positive), and 0 for SCC*mec* (negative) (Table 2). Sequencing of the SCC*mec* element compared to a reference SCC*mec* II sequence (S. aureus strain N315, GenBank accession number D86934.2) revealed the insertion of an ∼12-kb truncated arginine catabolic mobile element (ACME) (16) between *orfX* and SCC*mec* (Fig. 1). The insertion prevented amplification of the *orfX*-SCC*mec* target region because the forward and reverse primer sites were now ∼12 kb apart. Thus, the *C_T_* value of SCC*mec* target was 0.

### SCC insertion element 2: SCC_M1_.

Xpert MRSA/SA BC 2013 test results from a positive blood culture bottle were reported as MRSA negative and S. aureus positive, while the isolate (16445) recovered from the bottle was phenotypically susceptible to cefoxitin by both MIC and disk diffusion testing, although it was resistant to oxacillin by both MIC and disk diffusion testing (Table 1). The *C_T_* values for the Xpert test were 17.5 for *spa* (positive), 17.6 for *mec* (positive), and 0 for SCC*mec* (negative) (Table 2). The SCC*mec* element compared to reference sequence S. aureus strain M03-68 SCC*mec* IVg element (GenBank accession number DQ106887.1) revealed the insertion of an ∼14-kb SCC_M1_ element (17) adjacent to *orfX* and upstream of SCC*mec* IV(2B) (Fig. 1). The insertion of the SCC_M1_ element prevented detection of the SCC*mec* target in a manner similar to the insertion of the ACME described above, i.e., by separating the sequences targeted by the primers by ∼14 kb.

### SCC insertion element 3: SCC6838-like element.

Xpert MRSA/SA BC 2013 performed on blood culture isolate 15100 returned a result of MRSA negative and S. aureus positive, with *C_T_* values of 17.7 for *spa* (positive), 17.8 for *mec* (positive), and 0 for SCC (negative). The isolate was phenotypically MSSA by disk diffusion testing. However, because the Xpert test result was *mecA* positive, a cefoxitin induction test was performed on the isolate and MRSA colonies were recovered from inside the zone of inhibition (18) (Table 1). The Xpert MRSA/SA BC 2013 test result performed on the MRSA colony was still MRSA negative and S. aureus positive, with an SCC*mec C_T_* value of 0 (Table 2). Sequencing of the SCC*mec* element using MRSA strain N315 (GenBank accession number D86934.2) as a reference revealed the insertion of an ∼10-kb element between *orfX* and the reference SCC*mec* type II region. The insertion was a truncated version of the SCC_6838_ element (19) (designated ΨSCC_6838_), which separated the forward and reverse SCC*mec* primers by approximately 10 kb, preventing amplification of the SCC*mec* target.

### *spa* gene variant MRSA.

Xpert MRSA/SA BC2013 results on the positive blood culture bottle containing Gram-positive cocci in clusters were MRSA negative and S. aureus negative. The *C_T_* values for the test were 0 for *spa* (negative), 14.7 for *mec* (positive), and 15.9 for SCC*mec* (positive) (Table 2). The isolate (16514) recovered from the bottle was phenotypically MRSA by both MIC and disk diffusion testing (Table 1). Genomic analysis and comparison to the *spa* region of the reference S. aureus NRS384 genome (USA300 strain, GenBank accession number CP027476.1) revealed a deletion of 23 bp in the *spa* gene that prevented binding of the probe and precluded amplification of the *spa* target by the Xpert MRSA/SA BC 2013 test (Fig. 1).

### MSSA empty-cassette strain.

An Xpert MRSA/SA BC2013 test performed on a blood culture isolate returned a result of MRSA negative and S. aureus positive. The *C_T_* values were 21.1 for *spa* (positive), 0 for *mec* (negative), and 22.6 for SCC*mec* (positive) (Table 2). The isolate (15077) was phenotypically MSSA by both MIC and disk diffusion testing (Table 1), which was consistent with the PCR genotype (*mec* negative). Although the genotype and phenotype were concordant, sequencing and analysis of the SCC*mec* element were undertaken to understand the extent of the *mecA* deletion. The reference for SCC*mec* type II was S. aureus strain N315 (GenBank accession number D86934.2). Isolate 15077 revealed the total absence of *mecA* sequence, but there were remnants of an SCC*mec* element, specifically a sequence matching the *orfX*-SCC*mec* junction type ii, as reported by Hill-Cawthorne et al. (20). To further characterize the deletion, we compared the *orfX*-SCC_M1_ junction of strain 15077 to that of strain 15050, which was also an MSSA of *spa* type t002 and multilocus sequence type 6 (ST-5) and contained an SCC_M1_ insertion. Although similar in sequence, the *orfX*-SCC*mec* junction type ii sequence was not found in strain 15050 (Fig. 1), indicating that the deletions that generated the empty cassette were different from those in these otherwise similar strains.

### Algorithm change.

The Xpert MRSA/SA BC 2019 test with the new rule-based algorithms for analyzing the *C_T_* values for each of the three targets was performed on the six organisms described above. The results are shown in Table 2. For each of the organisms, the genotype reported with the Xpert MRSA/SA BC 2019 test was consistent with its oxacillin antimicrobial susceptibility test phenotype.

### Surveillance study.

Table 3 shows the genetic characterization of the 252 phenotypically MSSA isolates from hospitals across the United States and Europe. The distributions of the genetic alterations in the S. aureus isolates from the United States and selected countries in Europe are presented in Tables 4 and 5, respectively, and in Tables S1 and S2 in the supplemental material. Although all the isolates were phenotypically oxacillin susceptible, two were positive for *mec*A by Xpert MRSA/SA BC 2013, i.e., oxacillin-susceptible MRSA (OS-MRSA) (21). Both isolates yielded oxacillin-resistant colonies when grown in the presence of cefoxitin.

**TABLE 3:** Characterization of genetic alterations in 252 phenotypically methicillin-susceptible S. aureus isolates from the United States and Europe using Xpert MRSA/SA BC 2013 test

Phenotype	Genetic alteration	No. (%) of strains in which the alteration was detected
MSSA	No alterations detected	234 (92.9)
MSSA	Empty cassette	9 (3.6)
MSSA	SCC_M1_ insertion	4 (1.6)
MSSA	Empty cassette, *spa* variant	1 (0.4)
MSSA	Empty cassette with SCC_M1_ insertion	1 (0.4)
MSSA	*spa* variant	1 (0.4)
Oxacillin-susceptible MRSA	SCC*mec* variant	1 (0.4)
Oxacillin-susceptible MRSA	No alterations detected	1 (0.4)

**TABLE 4:** Genetic alterations identified in 152 methicillin-susceptible S. aureus isolates collected in the United States

State	No. of isolates						
	MSSA, no alterations detected	MSSA, empty cassette	Oxacillin-susceptible MRSA	Oxacillin-susceptible MRSA, SCC*mec* variant	MSSA with SCC_M1_ insertion	MSSA, empty cassette, with SCC_M1_ insertion	Total
NY	7	1	1		1		10
NJ	6				1		7
WA	4				1		5
NC	3			1			4
MN	3	1					4
MA	3	1					4
OR		1				1	2
Other states	116						116
Total	142	4	1	1	3	1	152

**TABLE 5:** Genetic alterations identified in 152 methicillin-susceptible S. aureus isolates collected in Europe

Country	No. of isolates					
	MSSA, no alterations detected	MSSA, empty cassette	MSSA, empty cassette, *spa* variant	MSSA, *spa* variant	MSSA with SCC_M1_ insertion	Total
Germany	11				1	12
France	9			1		10
Italy	9	1				10
Ireland	5	1				6
Russia	4	1	1			6
Portugal	2	2				4
Other countries	52					52
Total	92	5	1	1	1	100

There were 11 empty-cassette strains in total (4.4% of isolates) for which an SCC*mec* element or remnant sequences were present by sequence analysis but lacked the *mecA* gene (Table 3). Two also had additional genetic alterations (i.e., insertion of SCC_M1_ or a *spa* deletion). Among the empty cassette strains from the United States, two were from Oregon (both *spa* type t002), and one each was obtained from Massachusetts (*spa* type t121), Minnesota (*spa* type t922), and New York (*spa* type t5500) (data not shown). One of the empty-cassette isolates from Oregon was also positive for the SCC_M1_ element and further characterized by WGS (strain 15077) (Tables 1 to 4 and Fig. 1). For the European MSSA isolates, two with empty cassettes were obtained from Portugal (*spa* types t008 and t174), one was from Italy (an undefined *spa* type), one was from Ireland (t022), and one was from Russia (t127). Two MSSA isolates with mutations in *spa* were also identified: one was obtained from France and the other from Russia. The latter also had an empty cassette, suggesting a massive deletion of genetic material (not further characterized). *spa* types could not be established for either of the two isolates due to the genetic alterations affecting the variable Xr region of the *spa* gene (data not shown). Four isolates containing an SCC_M1_ element were identified (1.6% of all isolates tested, 2% if the empty cassette with SCC_M1_ is included). These were from New Jersey, New York, Washington, and Germany. All the isolates were tested with Xpert MRSA/SA BC 2019, which correctly identified all the MRSA isolates. However, two MSSA isolates with *spa* deletions were reported as MRSA negative, S. aureus negative, since the all targets in the test were negative.

## DISCUSSION

Although molecular diagnostic tests can provide rapid answers to guide therapeutic decisions for positive blood cultures that contain Gram-positive cocci in clusters, S. aureus strains containing a variety of genetic variations, such as insertions, deletions, and mutations within target sequences, can affect the accuracy of results (19, 20, 22–25). In this study, we noted a diverse set of genetic insertions leading to an MRSA-negative, S. aureus-positive result with Xpert MRSA/S BC 2013 before the new algorithms were introduced. The first three cases were isolates of MRSA in which the *orfX*-SCC*mec* junction sequence was altered by insertions of additional genetic elements using the same attachment site as SCC*mec* to integrate in *orfX*, as previously described (16, 23). This prevented the formation of PCR products. Interestingly, all three insertions were unique. The first was a truncated type II ACME (Δ ACME II) similar to the one described by Shore and colleagues; however, our isolate contained the *arc* gene cluster but not the *opp* gene cluster (16). The ACME has been reported previously for coagulase-negative staphylococci (CoNS) (26) and for the MRSA pulsed-field gel electrophoresis type USA300, where it is located downstream of SCC*mec* type IV (27). In our case, the Δ ACME II is followed by an ST-5-like SCC*mec* type II. A similar strain was described by Urushibara and colleagues (28). Additionally, the CI region observed in this study did not harbor a truncated J1 region of SCC*mec* type I (ΔJ1 SCC*mec* type I) between the ACME and SCC*mec* or immediately after *orfX*, as reported in the above-mentioned studies (16, 28). It has been hypothesized that the presence of an ACME adjacent to *orfX* and upstream of SCC*mec* could indicate integration of ACME into the chromosome prior to acquisition of SCC*mec* (16).

In the second case, we identified an SCC_M1_ element downstream of *orfX* and upstream of SCC*mec* type IV, similar to those described in prior studies (17, 29). Screening of 252 MSSA isolates from the United States and Europe identified only five isolates with SCC_M1_ insertions; one MSSA isolate was from Germany and four isolates were from the United States. Sequencing of all SCC_M1_-positive S. aureus isolates showed the element inserted directly after *orfX* in MSSA strain 15050 (and in 2 additional strains), in MRSA strain 16445, and in an empty-cassette strain (15077), suggesting that acquisition of this element can occur in *mecA*-positive as well as *mecA*-negative strains. Apparently, excision of SCC*mec* can occur independently of SCC_M1_. The primers used to screen our convenience sample for the presence of SCC_M1_ elements did not differentiate between SCC_M1_ and SCC_266_ (19); however, analysis of published SCC_266_ and SCC_M1_ sequences (GenBank accession numbers AB774374.1 and HE858191.1, respectively) showed that SCC_266_ elements contain an IS*431* element, which is not present in SCC_M1_. All the SCC_M1_/SCC_266_ elements identified in this study do not contain IS*431*, so they are likely SCC_M1_.

The genetic element identified in isolate 15100 carried a class 5 cassette chromosome recombinase (*ccr*) and partially matched the SCC_6838_ element described by Zhang et al. (19). However, in our case, this element preceded SCC*mec* type II, rather than a type I.

Isolates with SCC*mec* variants are reported by the Xpert MRSA/SA BC 2013 test as MRSA negative and S. aureus positive and could potentially lead to undertreatment of a patient until standardized phenotypic susceptibility testing results become available. However, not every oxacillin-susceptible phenotypic test result is accurate (18, 21). During this study, we encountered three S. aureus isolates that were initially reported as susceptible to cefoxitin or oxacillin but expressed methicillin resistance once exposed to cefoxitin. This phenomenon, often referred to generically as induction, was recently shown by Goering et al. (30) to be a result of mutations in *mecA* that restore the MRSA phenotype by repairing stop codons or missense mutations.

In isolate 16514, a 23-bp deletion in the *spa* gene caused an MRSA-negative, S. aureus-negative result with the Xpert MRSA/SA BC 2013 test because a positive *spa* result is required for S. aureus identification. This isolate was reported correctly as MRSA positive by the Xpert MRSA/SA BC 2019 test. Deletions and rearrangements in the *spa* region, although rare, have been reported as the cause of failed *spa* typing. For example, a 2009 study by Baum and colleagues reported that 4.7% of MSSA and 0.7% of MRSA strains that failed *spa* typing did so because of deletions that ranged between 161 and 705 bp and in two cases (0.1% of isolates tested) encompassed the entire *spa* gene (31). Deletions in *spa* have been observed among MRSA strains from inpatients in hospitals receiving antibiotics, suggesting that antibiotic pressure may contribute to these changes (22, 32, 33). In contrast, our survey of S. aureus isolates in the United States and Europe identified only two isolates with mutations in the *spa* gene, constituting only 0.8% of the isolates tested, suggesting that this is a rarer phenomenon among MSSA than reported previously. This may be due in part to the fact that we only tested blood isolates, for which having mutations in this major virulence factor may place strains at a selective disadvantage for survival (34).

Empty-cassette S. aureus strains occur when *mecA* is deleted from the SCC*mec* element but portions of SCC*mec* remain in the *attB* site within *orfX*. Such isolates are usually reported correctly as MRSA negative and S. aureus positive both by the Xpert MRSA/SA BC 2013 test and by the Xpert MRSA/SA BC 2019 test. However, a false-positive MRSA result may occur if a methicillin-resistant coagulase-negative staphylococcus (CoNS), such as Staphylococcus epidermidis, is present in the same positive blood culture vial as an empty-cassette S. aureus strain. The presence of *mecA* from CoNS, combined with the *spa* and SCC*mec* from S. aureus, can yield a discordant result of MRSA (23), although we did not encounter this combination of organisms in our study.

In summary, while a variety of genetic alterations can occur in S. aureus isolates that impact the results of molecular tests, none of these appear to be common in either the United States or Europe. A limitation of our study is that we focused only on MSSA isolates in the surveillance study because we were trying to identify OS-MRSA isolates, empty-cassette strains, and those with insertion elements, particularly SCC_M1_, in the *orfX* region. Testing of MRSA isolates may have identified additional strains with genetic alterations. Nonetheless, the new rule-based algorithms of the Xpert MRSA/SA BC 2019 test provided correct results for MRSA isolates with *spa* variants or SCC*mec* variants, including the three types of genetic insertions noted here.

## Supplementary Material

Supplemental file 1
