# Supplementary material for: Updating Molecular Diagnostics for Detecting Methicillin-Susceptible and Methicillin-Resistant Staphylococcus aureus Isolates in Blood Culture Bottles
Source: J Clin Microbiol. 2019 Oct 23;57(11):e01195-19. doi: 10.1128/JCM.01195-19 (PMC6813022; doi:10.1128/JCM.01195-19)
Supplement: Supplemental file 1 [file JCM.01195-19-s0001.pdf]

Table S1. Genetic alterations identified in 152 methicillin-susceptible *S. aureus* isolates collected in the United States

State	MSSA, no alterations detected	MSSA Empty cassette	Oxacillin-susceptible MRSA	Oxacillin-susceptible MRSA, SCC*mec* variant	MSSA with SCC_{M1} insertion	MSSA Empty cassette with SCC_{M1} insertion	Total
TX	11						11
NY	7	1	1		1		10
CA	10						10
NJ	6				1		7
IL	7						7
AZ	6						6
FL	6						6
WI	6						6
KY	5						5
MO	5						5
WA	4				1		5
IA	4						4
OH	4						4
VA	4						4
CO	4						4
PA	4						4
NC	3			1			4
MN	3	1					4
AL	4						4
LA	4						4
MA	3	1					4
MI	4						4
IN	3						3
GA	3						3
UT	2						2
TN	2						2
DE	2						2
NM	2						2
AK	2						2
AR	2						2
NE	2						2
VT	2						2
ND	2						2
OR		1				1	2
ME	2						2
MD	1						1
KS	1						1
**Total**	**142**	**4**	**1**	**1**	**3**	**1**	**152**

Table S2. Genetic alterations found in 100 methicillin-susceptible *S. aureus* isolates collected in Europe

Country	MSSA, no alterations detected	MSSA Empty cassette	MSSA Empty cassette, *spa* variant	MSSA, *spa* variant	MSSA with SCC_{M1} insertion	Total
Germany	11				1	12
France	9			1		10
Italy	9	1				10
United Kingdom	10					10
Spain	8					8
Ireland	5	1				6
Russia	4	1	1			6
Sweden	5					5
Hungary	4					4
Portugal	2	2				4
Romania	4					4
Turkey	4					4
Belarus	3					3
Belgium	3					3
Czech Republic	3					3
Greece	3					3
Poland	3					3
Slovenia	2					2
**Total**	**92**	**5**	**1**	**1**	**1**	**100**
